# NUF2 Drives Clear Cell Renal Cell Carcinoma by Activating HMGA2 Transcription through KDM2A-mediated H3K36me2 Demethylation

**DOI:** 10.7150/ijbs.70972

**Published:** 2022-05-16

**Authors:** Jiatian Lin, Xiangling Chen, Hongjian Yu, Shasha Min, Yequn Chen, Zesong Li, Xina Xie

**Affiliations:** 1 Department of Minimally Invasive Intervention, Peking University Shenzhen Hospital, Shenzhen 518000, Guangdong, China; 2 Guangdong Provincial Key Laboratory of Systems Biology and Synthetic Biology for Urogenital Tumors, Shenzhen Key Laboratory of Genitourinary Tumor, Department of Urology, The First Affiliated Hospital of Shenzhen University, Shenzhen Second People's Hospital (Shenzhen Institute of Translational Medicine), Shenzhen, 518000, Guangdong, China; 3 Institute of Precision Medicine, Peking University Shenzhen Hospital, Shenzhen 518000, Guangdong, China; 4 Department of Community Surveillance, The First Affiliated Hospital of Shantou University Medical College, Shantou 515041, Guangdong, China

**Keywords:** Clear cell renal cell carcinoma, NUF2, HMGA2, KDM2A, H3K36me2

## Abstract

The poor sensitivity of clear cell renal cell carcinoma (ccRCC) to conventional chemotherapy and radiotherapy makes its treatment challenging. The Ndc80 kinetochore complex component (NUF2) is involved in the development and progression of several cancers. However, its role in ccRCC remains unclear. In this study, we investigated the biological functions and underlying mechanism of NUF2 in ccRCC. We found that NUF2 expression was increased in ccRCC and associated with poor prognosis. Altering NUF2 level affected cell proliferation, migration, and invasion. Moreover, NUF2 acted as a potential oncogene to promote the progression of ccRCC through epigenetic activation of high-mobility group AT-hook 2 (HMGA2) transcription by suppressing lysine demethylase 2A expression and affecting its occupancy on the *HMGA2* promoter region to regulate histone H3 lysine 36 di-methylation modification. In addition, Kaplan-Meier and multivariate analysis revealed that patients whose NUF2 and HMGA2 were both elevated showed the shortest survival; and the number of upregulated markers acted as an independent predictor to evaluate survival probability. Thus, our results demonstrate that NUF2 promotes ccRCC progression, at least partly by epigenetically regulating HMGA2 transcription, and that the NUF2-HMGA2 axis could be an ideal therapeutic target and a promising prognostic indicator for ccRCC.

## Introduction

Renal cell carcinoma is the most common malignancy of kidney cancer. Eighty to ninety percentage of all renal cell carcinoma are clear cell renal cell carcinoma (ccRCC) 1. While localized or locally advanced renal cell carcinoma can be surgically resected, approximately 30% of patients show local recurrence or distant metastasis. Even with advanced care, the 5-year survival rate of patients with metastatic ccRCC is only 10% 2, 3. The poor prognosis of patients with advanced disease might be due to the poor sensitivity of ccRCC to conventional radiotherapy and chemotherapy 4, 5. Therefore, there is an urgent need to improve our understanding of the mechanisms underlying ccRCC pathogenesis and identify novel, sensitive, and reliable molecular markers that can serve as promising prognostic indicators and therapeutic targets for ccRCC 6.

It is well known that abnormal chromosome separation during mitosis is a common cause of carcinogenesis and that the kinetochore connected to spindle microtubules is essential for accurate chromosome segregation during mitosis 7-10. The Ndc80 kinetochore complex component (NUF2), also known as cell division cycle associated 1, is a key molecule that stabilizes spindle microtubule-kinetochore attachment during the metaphase of cell division 11, 12. DeLuca *et al*. found that in HeLa cells, after knockdown of NUF2, spindle formation occurred normally, but kinetochores failed to connect with spindle microtubules, which caused aberrant chromosome segmentation and induced mitotic cells to undergo apoptosis 13. Therefore, it is not surprising that dysregulation of NUF2 expression and function can promote tumor development. For example, NUF2 is reported to be overexpressed in gastric cancer, colon cancer, and pancreatic cancer, and depletion of NUF2 has been shown to suppress tumor cell proliferation 14, 15. However, very few studies have investigated the association between NUF2 and ccRCC. We previously reported that NUF2 is involved in long noncoding RNA CDKN2BAS1-mediated malignancy of ccRCC 16. However, little is known about the specific function and clinical implications of NUF2 and its underlying mechanism in ccRCC.

In this study, we investigated the molecular functions and their implications and the clinical value of NUF2 in ccRCC. We used The Cancer Genome Atlas (TCGA) and ONCOMINE databases to predict the expression level of NUF2 in ccRCC. Our analyses showed that elevated NUF2 expression correlates with poor clinical outcomes, and NUF2 was an independent prognostic indicator for ccRCC. Our *in vitro* experiments showed that NUF2 promoted proliferation, migration, and invasion of ccRCC cells by regulating the oncogene high-mobility group AT-hook 2 (*HMGA2*). We also comprehensively explored the value of the NUF2-HMGA2 axis as a prognostic biomarker in ccRCC.

## Materials and Methods

### Computational analysis and database

The RNA expression of NUF2 in ccRCC tissues and normal tissues was profiled based on normalized RNA-sequencing data in the TCGA-ccRCC dataset (TCGA_ccRCC_exp_HiSeqV2-2015-02-24) from the UCSC Xena Browser (https://xenabrowser.net/datapages/) and was further analyzed using the ONCOMINE gene expression array datasets (www.oncomine.org)^17^.

### Clinical tissue samples

Sixty-seven pairs of ccRCC tissues and matched adjacent normal tissues were collected from patients admitted to the First Affiliated Hospital of Shantou University Medical College. All participants provided written informed consent. The study was conducted in accordance with the Declaration of Helsinki, and the protocol was approved by the Ethics Committee of the First Affiliated Hospital of Shantou University Medical College (number: 2020-031). All tissue samples were obtained directly from surgery after tissue removal for routine pathological examination and confirmed for ccRCC. All tissue samples were immediately flash-frozen in liquid nitrogen and subsequently stored at -80 ℃ for RNA analysis.

### Cell culture and treatment

The human renal tubular epithelial cell line HK-2 and ccRCC cell lines ACHN, 786-O, and 769-P were obtained from the American Type Culture Collection (ATCC, Rockville, MD, USA). HK-2 cells were cultured in DMEM/F12 medium (Gibco, USA.). ACHN cells were cultured in Eagle's MEM medium (Gibco, USA), and 786-O and 769-P cells were cultured in RPMI-1640 medium (Hyclone, USA). All media were supplemented with 10% fetal bovine serum (FBS; Gibco, USA), and all cell lines were cultured in a humidified incubator at 37 ℃ with 5% CO_2_.

Small interfering RNAs (siRNAs) for NUF2 and lysine demethylase 2A (KDM2A) were obtained from RiboBio Co. (Guangzhou, China) and transfected at a final concentration of 60 nM using Lipofectamine RNAiMAX (No.13778-150, Invitrogen, USA), following the manufacturer's instructions. The pcDNA3.1-NUF2 plasmid was obtained from Transheep (Shanghai, China) and transfected at a final concentration of 5.0 μg/mL using FuGENE^®^ HD Reagent (No. E2311, Promega, Madison, WI, USA) following the manufacturer's instructions. The lentiviral vectors used to overexpress HMGA2 were constructed by GenePharma Co. (Shanghai, China). Human full-length cDNA of HMGA2 was cloned into the expression vector LV5-EF-1a-GFP-Puro, named LV5-HMGA2, and the empty lentiviral expression vector was used as a control (LV5-Vector). The ccRCC cells were plated onto six-well plates and transfected with the lentiviral vector according to the manufacturer's instructions. siRNA sequences were as follows: siRNA-NUF2-1: 5′-GGACTCCTATGCTAAGATA-3′; siRNA-NUF2-2: 5′-GCATGAAGATGTTAAGCAA-3′; siRNA-NUF2-3: 5′-GGAGGACCAAATTGAGAGT-3′; and siRNA-KDM2A: 5′-GCATGGATTTGGAGTTAAA-3′.

### qRT-PCR analysis

Total RNA was extracted from ccRCC tissues and cells using Trizol (No. 15596018, Invitrogen, USA), followed by reverse transcription using the PrimeScript RT reagent Kit with gDNA Eraser (No. RR047A, Takara Bio, Inc., Japan) for cDNA synthesis and genomic DNA removal. qRT-PCR was performed using a QuantiNova^TM^ SYBR Green PCR mix kit (No. 04933914001, Roche, Switzerland) and was carried out in the Applied Biosystems Prism 7500. The relative expression levels of NUF2 were compared to that of β-actin, and fold-changes were calculated using the 2^-∆∆Ct^ method for cell experiments and the 2^-∆Ct^ method for tissue samples 18. The primers used were as follows: NUF2 forward, 5′-GGAAGGCTTCTTACCATTCAGC-3′ and reverse, 5′-GACTTGTCCGTTTTGCTTTTGG-3′; HMGA2 forward, 5′-ACCCAGGGGAAGACCCAAA-3′ and reverse, 5′-CCTCTTGGCCGTTTTTCTCCA-3′; β-actin forward, 5′-CTGGAACGGTGAAGGTGACA-3′ and reverse, 5′-AAGGGACTTCCTGTAACAATGCA-3′.

### Western blot analysis

Cell lines were lysed in RIPA buffer with 1% protease inhibitor cocktail (No. 539134-1SET, Millipore, USA). Total protein concentration was determined using a Pierce™ BCA Protein Assay Kit (No. 23227, Thermo Fisher Scientific, Inc.). The cell lysate (30 µg total protein) was subjected to 8% SDS-PAGE and transferred to PVDF membranes (Millipore, USA). Immunoblotting was performed using primary antibodies against NUF2 (1:800; No. 15731-1-AP; Proteintech, USA), E-cadherin (1:1000; No. 3195S; Cell Signaling Technology, USA), HMGA2 (1:1000; No. 20795-1-AP; Proteintech, USA), KDM2A (1:1000; No. ab191387; Abcam, England), polycomb group factor 6 (PCGF6; 1:1000; No. 24103-1-AP; Proteintech, USA), and GAPDH (1:3000; No. 60004-1-Ig; Proteintech, USA). Corresponding secondary antibodies were applied, and blots were developed using the SuperSignalTM West Dura Extended Duration Substrate reagent (Thermo Fisher Scientific, Inc.) with an Amersham Imager 600 (GE Healthcare).

### Immunohistochemistry analysis

Paraffin-embedded ccRCC tissue microarray (No. HkidE180Su03) was purchased from Shanghai Biochip Company Ltd. (Shanghai, China). The sections were dewaxed in xylene and rehydrated with different concentrations of ethanol. Endogenous peroxidase was blocked with 3% H_2_O_2_, and microwave heating was performed for antigen retrieval. After blocking nonspecific antigen binding with 3% BSA at 25 °C for 1 h, the sections were incubated with a specific primary antibody against NUF2 (1:200; No. 15731-1-AP; Proteintech, USA) at 4 °C overnight. After incubation with the HRP-conjugated secondary antibody at 37 °C for 1 h, the sections were counterstained with hematoxylin and stained with diaminobenzidine. Images were taken using a Nikon ECLIPSE Ti microscope (Fukasawa, Japan). The immunostaining intensity of each sample was graded as follows: negative = 0, weak = 1, moderate = 2, or strong = 3. The proportion of positively stained cells was graded as negative = 0, 0%-25% = 1, 26%-50% = 2, 51%-75% = 3, or 76%-100% = 4. The immunostaining score was calculated as the sum of the intensity and positive rate scores, and was graded as follows: 0 score = negative (-), 1-3 scores = weakly positive (+), 4-5 scores = moderate positive (++), or 6-7 scores = strongly positive (+++).

### Colony formation assay

Transfected cells were seeded in 6-well plates at a density of 2000 cells/well. After 10-14 days, cell colonies were fixed with 4% paraformaldehyde, air-dried, and stained with crystal violet (No. C0121-100ml, Beyotime, Shanghai, China). The colonies were imaged and counted.

### Migration and invasion assays

Cell migration and invasion abilities were measured using transwell chambers (No. 3422, 8-μm pore size, BD Biosciences, USA) following the manufacturer's protocol. For the migration assay, 5 × 10^4^ transfected cells cultured in 0.5% FBS medium were seeded onto the upper chambers of the transwell, and medium with 20% FBS was added to the lower chamber. After 18 h of incubation, the chamber was fixed with 4% paraformaldehyde and stained with crystal violet (Beyotime, Shanghai, China). Cells that migrated through the pores were imaged and counted. For the invasion assay, the membrane was precoated with matrigel, and 1.0 × 10^5^ transfected cells were added to the top chamber.

### RNA sequencing analysis

After transfecting 769-P and ACHN cells with siRNA-NUF2 or siRNA-control (siRNA-NC) for 48 h, total RNA from each group was extracted and sequenced on the Illumina Novaseq 6000 platform at Novogene Co. Ltd. RNA sequencing results were read in FASTA format after fastp data quality evaluation and filtering. DESeq2 was used to analyze differentially expressed genes (DEGs) with cutoff values of *p* adj-value < 0.05, and |log_2_ (fold change) | > 0. The RNA sequencing data were uploaded to the GEO database (GSE192754).

### Chromatin immunoprecipitation assays

Chromatin immunoprecipitation (ChIP) was performed with EZ-Magna ChIP A/G Kits (Millipore, USA) according to the manufacturer's protocol. ChIP grade antibodies were as follows: anti-KDM2A (No. ab191387; Abcam, England), anti-RNA Polymerase II (RNA Pol II; No. ab264350, Abcam, England), anti-Histone H3 lysine 36 mono methylation (H3K36me; No. ab9048, Abcam, England), anti-Histone H3 lysine 36 di-methylation (H3K36me2; No. ab9049, Abcam, England), anti-Histone H3 lysine 36 trimethylation (H3K36me3; No. ab9050, Abcam, England), and normal IgG (Millipore, USA). Immunoprecipitated DNA was analyzed by qRT-PCR, and normalized to the input DNA. Primer sequences for the promoter region of *HMGA2* were as follows: forward, 5′- TTCCCACTCACAGTGAACCG -3′ and reverse, 5′- CTTTACCTGCGCCTCTACCG -3′.

### Statistical analysis

Analyses were performed using SPSS 23.0 (SPSS Inc., Chicago, IL, USA). The differences between groups for cells were analyzed using an independent sample* t*-test, one-way ANOVA, or Dunnett's multiple comparison test as appropriate. The expression of NUF2 in ccRCC tissues and matched adjacent normal tissues was analyzed using a paired-sample* t*-test. Survival curves were estimated using the Kaplan-Meier method and compared using log-rank tests. Survival data were evaluated using univariate and multivariate Cox regression analyses. All data are shown as the mean ± SEM. Statistical significance was defined as *p* < 0.05.

## Results

### Elevated expression of NUF2 correlated with poor prognosis in patients with ccRCC

**Figure 1:**
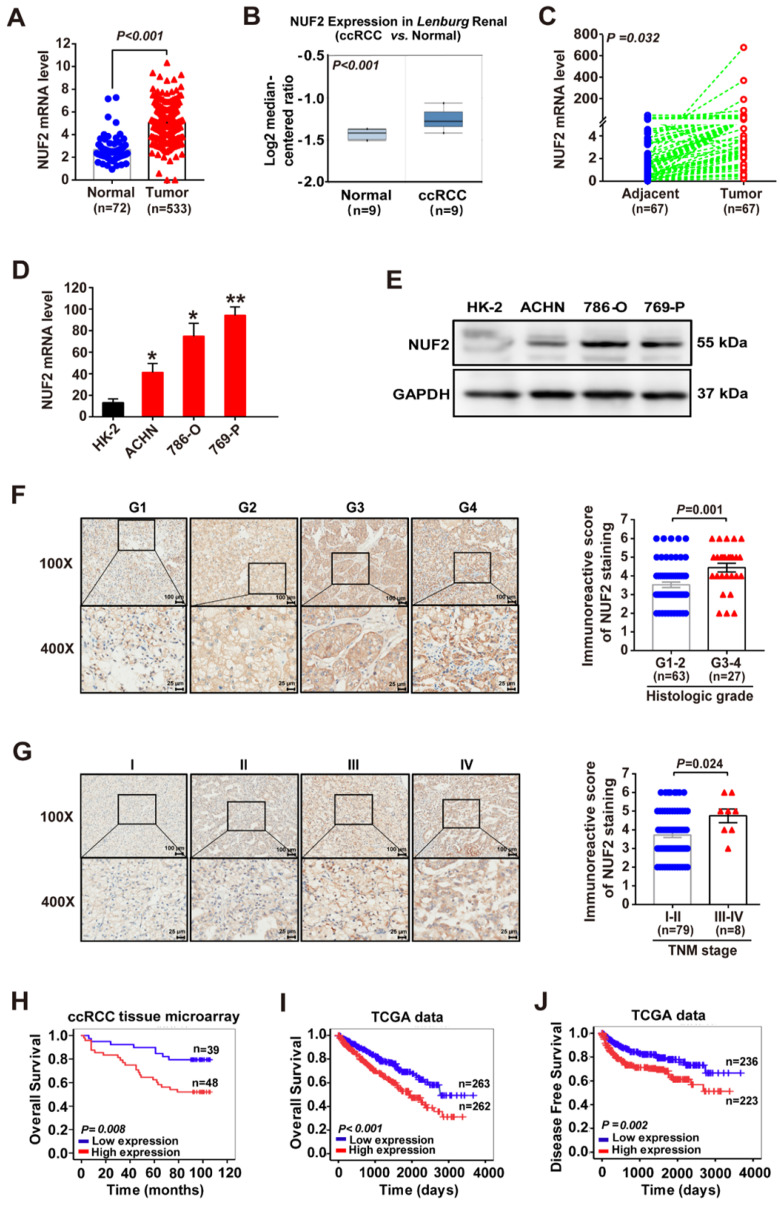
** NUF2 is upregulated in ccRCC and correlated with the poor prognosis of patients. (A)** Analysis of NUF2 mRNA levels in ccRCC tissues (n = 533) compared with normal tissues (n = 72) using RNA-sequencing data of TCGA dataset.** (B)** NUF2 mRNA level in ccRCC tissues and normal tissues from *Lenburg's* dataset in ONCOMINE database (n = 9). **(C)** qRT-PCR analysis of NUF2 expression in 67 paired ccRCC tissues (Tumor) and adjacent normal tissues (Adjacent); β-actin was used as the internal control.** (D, E)** qRT-PCR and western blot analysis of NUF2 mRNA and protein expression in ACHN, 786-O, 769-P, and HK-2, with β-actin or GAPDH as the internal control, respectively. Data in (D) are representative from three independent experiments. **(F, G)** Immunostaining for NUF2 in patients with a different histologic grade (F) and TNM stage (G) from the paraffin-embedded ccRCC tissue microarray (Scale bar: 100 μm or 25 μm; No. HkidE180Su03); the immunostaining score of NUF2 was calculated. **(H-J)** Kaplan-Meier analysis showed the overall survival (OS) and disease-free survival (DFS) in ccRCC patients based on the expression of NUF2; ccRCC tissues showed weakly positive immunostaining score were defined as the low expression group, and the moderate to strong positive samples were defined as the high expression group in (H); in (I, J), the high and low expression groups were divided by the median of NUF2 mRNA levels in TCGA dataset. Error bars represent the SEM.

We first compared NUF2 expression between ccRCC and normal tissues, using the patient cohort from the TCGA database, and found that the mRNA expression of NUF2 was significantly upregulated in ccRCC tissues (Figure 1A). A similar result was obtained on our analysis of *Lenburg*'s dataset 19 from the ONCOMINE database (Figure 1B). Next, qRT-PCR analysis of NUF2 in 67 pairs of ccRCC and matched tumor-adjacent tissues showed that NUF2 was significantly increased in tumor tissues (Figure 1C). Among that, NUF2 levels were upregulated in 94% (63/67) of patients with ccRCC (Figure S1). Consistent with the increase in clinical samples, elevated mRNA, and protein levels of NUF2 were also observed in ccRCC cell lines ACHN, 786-O, and 769-P, compared to the expression in normal renal tubular epithelial cell HK-2 (Figure 1D, 1E). These results suggest that NUF2 expression levels are increased in ccRCC tissues and cells.

Furthermore, the TCGA dataset revealed that elevated NUF2 expression was associated with large tumor size, higher histologic grades, advanced tumor node metastasis (TNM) stages, and metastases (Table S1). This was further confirmed by the higher immunoreactive score of NUF2 staining in the tissues of patients with higher tumor grades and advanced TNM stages (Figure 1F, 1G and Table S2). Moreover, the Kaplan-Meier survival curve showed that ccRCC patients with high NUF2 levels had significantly shorter overall survival (OS) and disease-free survival (DFS) rates than those with low NUF2 expression (Figure 1H-1J). In addition, age, tumor size, histological grade, tumor invasion, TNM stage, metastasis, and NUF2 mRNA and protein levels correlated with the patient survival (Table 1). The Cox regression multivariate analysis further demonstrated that NUF2 could serve as an independent prognostic factor for worse OS (Hazard Ratio (HR) = 1.020, 95% Confidence Interval (CI):1.002-1.039, *p* = 0.031 for tissue microarray dataset; HR = 1.280, 95% CI:1.101-1.488, *p* = 0.001 for TCGA dataset) and DFS (HR = 1.227, 95% CI:1.016-1.482, *p* = 0.034 for TCGA dataset) in ccRCC patients (Table 1). In summary, elevated NUF2 expression correlated with poor clinical outcomes in patients with ccRCC.

### NUF2 promoted ccRCC cell proliferation, migration, and invasion

**Figure 2:**
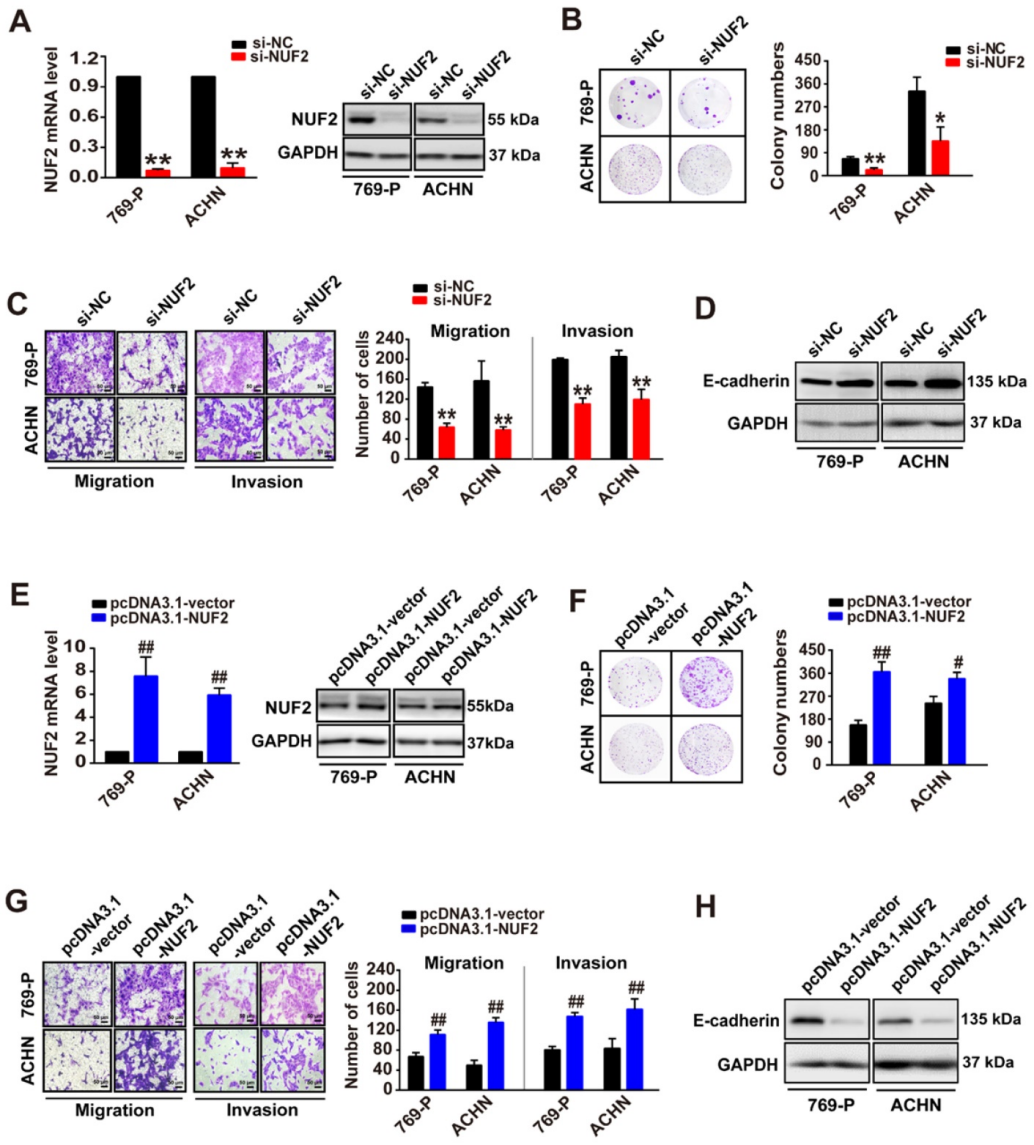
** NUF2 promotes ccRCC cell proliferation, migration, and invasion. (A, E)** qRT-PCR and western blot analysis of NUF2 mRNA and protein expression in 769-P and ACHN cells transfected with the si-NUF2 or pcDNA3.1-NUF2 for 48 h, with β-actin or GAPDH as the internal control, respectively. **(B, C, F, G)** Colony formation assays were used to evaluate cell proliferation in 769-P and ACHN cells transfected with si-NUF2 or pcDNA3.1-NUF2, respectively (B, F); transwell assays were performed to evaluate the cell migration and invasion (C, G); ImageJ software was used for cell counting. Scale bar: 50 μm.** (D, H)** Western blot analysis of E-cadherin protein expression in 769-P and ACHN cells transfected with si-NUF2 or pcDNA3.1-NUF2, with GAPDH as the internal control. Error bars represent the SEM from three independent experiments. **p < 0.01 *vs*. si-NC, *p < 0.05 *vs*. si-NC; ^##^*p* < 0.01 *vs*. pcDNA3.1-vector, ^#^*p* < 0.05 *vs*. pcDNA3.1-vector.

Since NUF2 levels were correlated with tumor size and poor clinical outcomes, we further investigated the role of NUF2 in ccRCC. A colony formation assay was performed following NUF2 knockdown with siRNA (si-NUF2, including siRNA2, and siRNA3 against NUF2; Figure S2). As expected, the knockdown of NUF2 using siRNA markedly decreased NUF2 mRNA and protein expression levels in 769-P and ACHN cells, resulting in a reduction in the number of colonies formed by ~70% and 60% in 769-P and ACHN cells, respectively (Figure 2A, 2B). In addition, transwell assays demonstrated that NUF2 suppression also considerably attenuated the migratory and invasive capacities of 769-P and ACHN cells, and accordingly led to an increase in the expression of E-cadherin (Figure 2C, 2D); the later as a classic marker of epithelial-mesenchymal transition, is specifically expressed in epithelial and its increased indicates the suppression of migration and invasion in tumor cell 20. Consistently, NUF2 overexpression plasmid (pcDNA3.1-NUF2), which significantly increased NUF2 mRNA and protein levels in ccRCC cells, generated opposite effects; overexpression of NUF2 could sharply augment cell proliferative, migratory, and invasive capacities (Figure 2E-2H). These data indicate that NUF2 acts as an oncogene that drives ccRCC cell proliferation, migration, and invasion.

### NUF2 involved in ccRCC progression via regulating HMGA2 expression

**Figure 3:**
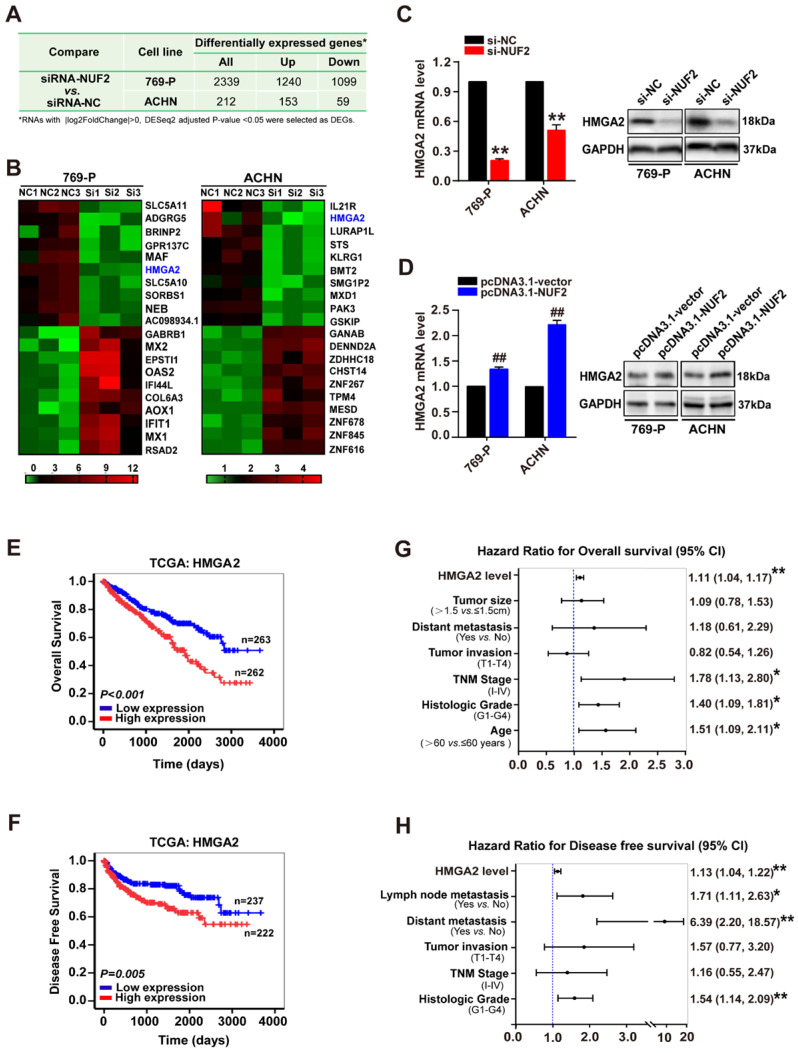
** NUF2 regulates HMGA2 expression. (A)** The differentially expressed genes (DEGs) from the RNA-sequencing in 769-P and ACHN cells transfected with siRNA-NUF2 or siRNA-NC, respectively. **(B)** Heat map of the top 10 DEGs that were upregulated and downregulated in 769-P and ACHN cells from RNA-sequencing, respectively. **(C, D)** qRT-PCR and western blot analysis of HMGA2 mRNA and protein expression in 769-P and ACHN cells transfected with the si-NUF2 (C) or pcDNA3.1-NUF2 (D) for 48 h, with β-actin or GAPDH as the internal control, respectively. Error bars represent the SEM from three independent experiments. **p < 0.01 *vs*. si-NC; ^##^*p* < 0.01 *vs*. pcDNA3.1-vector. **(E, F)** Kaplan-Meier analysis showed the overall survival (OS) and disease-free survival (DFS) in ccRCC patients based on the expression of HMGA2; the high and low expression groups were divided by the median of HMGA2 mRNA levels in TCGA dataset. **(G, H)** Multivariate Cox regression analysis of clinicopathologic variables and HMGA2 level associated with OS (G) and DFS (H) in patients with ccRCC in TCGA database, **p < 0.01, *p < 0.05.

Subsequently, to elucidate the underlying mechanism of NUF2 in ccRCC progression, RNA sequencing analysis was performed in ccRCC cells after knocking down NUF2 using siRNA. As shown in Figure 3A and Figure S3, a total of 2339 (1099 downregulated genes and 1240 upregulated genes) and 212 DEGs (59 downregulated genes and 153 upregulated genes) were identified between siRNA-NUF2 and the negative control groups (siRNA-NC) in 769-P and ACHN, respectively. Interestingly, among the top 10 DEGs that were downregulated and upregulated in 769-P and ACHN cells, *HMGA2* was the only downregulated gene in both 769-P and ACHN cells (Figure 3B). Next, qRT-PCR and western blot assays verified that depletion of NUF2 decreased HMGA2 mRNA and protein levels in 769-P and ACHN cells, and the opposite effect was observed in NUF2-overexpressed cells, indicating that *HMGA2* may be one of the target genes regulated by NUF2 in ccRCC (Figure 3C, 3D). Importantly, similar to NUF2, higher HMGA2 expression was associated with higher tumor grades, advanced TNM stages, metastases, and poor OS and DFS (Table S1, Figure 3E, 3F and Table S3). Cox regression multivariate analysis also revealed that HMGA2 could be an independent prognostic factor for shorter OS (HR = 1.11, 95% CI:1.04-1.17, *p* < 0.01; Figure 3G) and DFS (HR = 1.13, 95% CI:1.04-1.22, *p* < 0.01; Figure 3H) in ccRCC patients. Therefore, we hypothesized that NUF2 might promote ccRCC malignancy by regulating HMGA2 expression.

**Figure 4:**
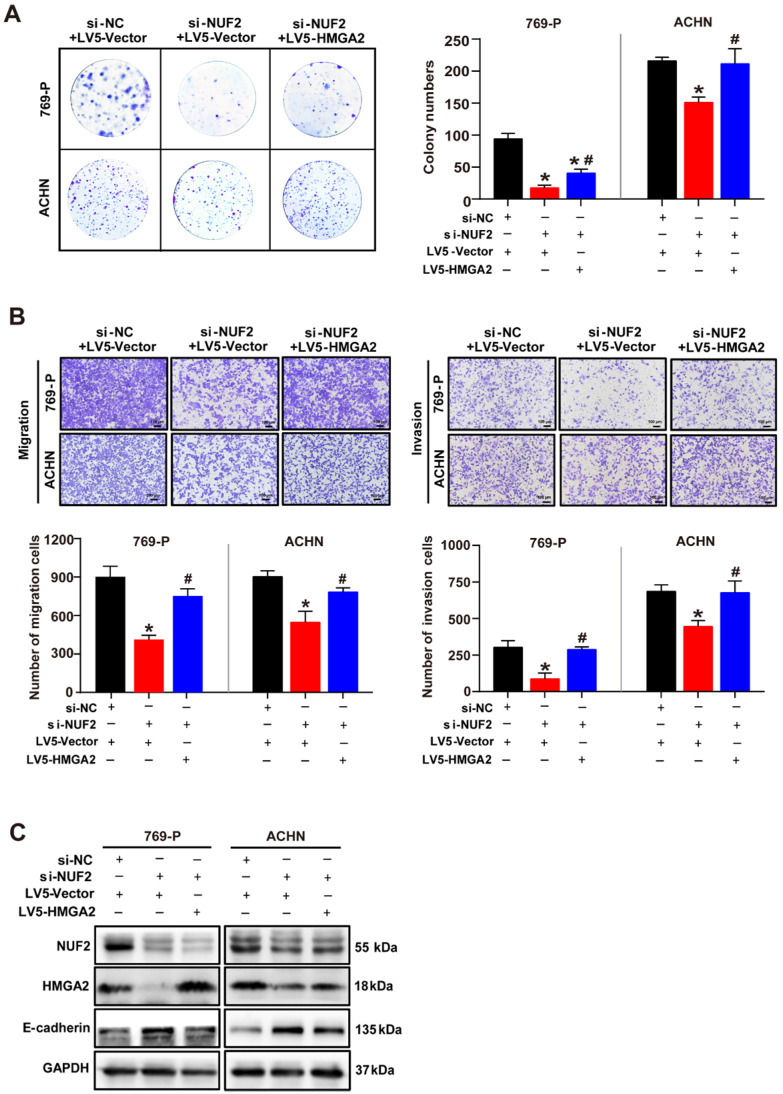
** Restored HMGA2 expression alleviates NUF2 depletion-mediated inhibition of proliferation, migration and invasion of ccRCC cells.** 769-P and ACHN cells were transfected with si-NUF2 plus LV5-HMGA2. **(A)** Colony formation assays were performed to assess cell proliferation. **(B)** Transwell assays were used to evaluate cell migration and invasion; ImageJ software was used for cell counting. Scale bar: 50 μm. **(C)** Western blot analysis of NUF2, HMGA2, and E-cadherin protein expression in 769-P and ACHN cells, with GAPDH as the internal control. Error bars represent the SEM from three independent experiments. *p < 0.05 *vs*. si-NC+LV5-Vector; ^#^*p* < 0.05 *vs*. si-NUF2+LV5-Vector.

To verify this hypothesis, HMGA2 was overexpressed through transient transfection with LV5-HMGA2 in ccRCC cells following NUF2 knockdown. As expected, ectopic HMGA2 expression significantly recovered the inhibitory effects of NUF2 depletion on cell proliferation, migration, and invasion. We observed that the number of colonies formed in 769-P and ACHN cells were attenuated by depleting NUF2, while both effects were prominently blocked after the restoration of HMGA2 expression (Figure 4A). Additionally, restoring HMGA2 levels also notably reversed the number of migrating and invading cells decreased by NUF2 depletion and recovered the expression of E-cadherin to basal levels (Figure 4B, 4C). Cumulatively, these data confirmed that NUF2 is involved in ccRCC malignancy, at least partly through the regulation of HMGA2, implying that the NUF2-HMGA2 axis is important for ccRCC progression.

### NUF2 activated *HMGA2* transcription by affecting the recruitment of KDM2A to regulate H3K36me2 modification in the promoter region

**Figure 5:**
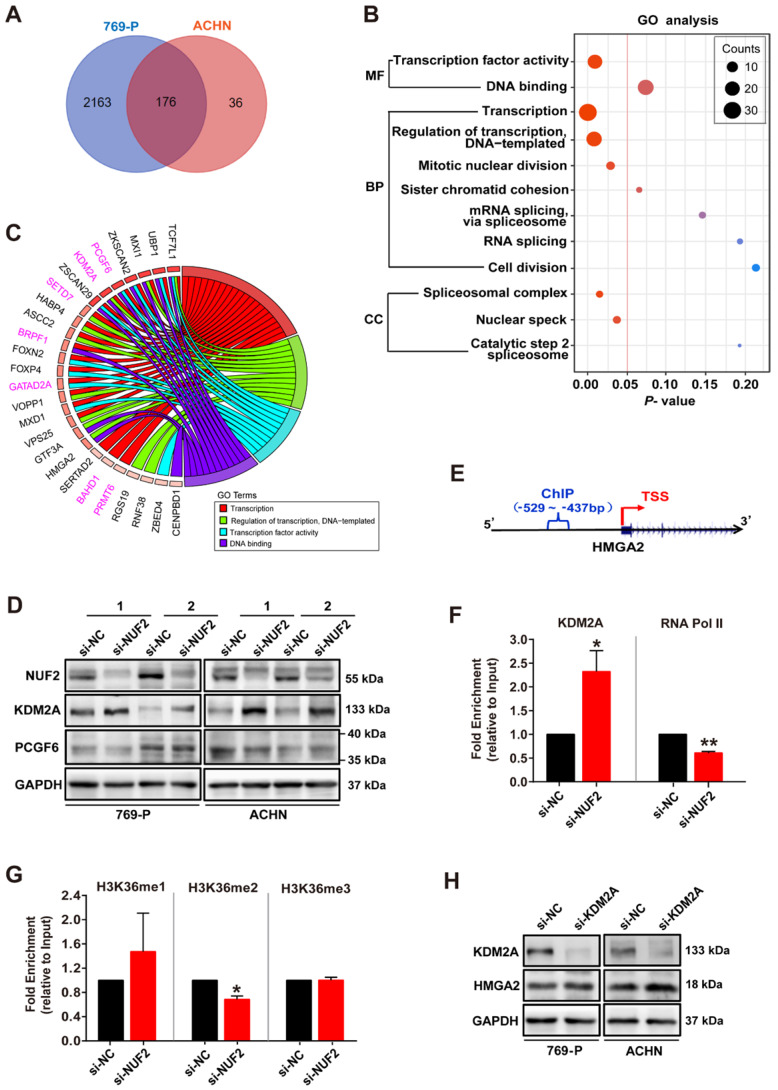
** NUF2 activates *HMGA2* transcription by affecting the recruitment of KDM2A to regulate H3K36me2 modification in the promoter region. (A)** Venn diagram showing 176 overlapping DEGs from the RNA-sequencing in 769-P and ACHN cells transfected with si-NUF2 or si-NC. **(B)** Gene ontology (GO) analysis of above 176 overlapping DEGs in 769-P and ACHN cells. MF, molecular function; BP, biological process; CC, cell component. **(C)** Chord diagram representing the GO terms related to transcriptional processes, including transcription, regulation of transcription, transcription factor activity, and DNA binding, paired with 26 genes specifically involved in transcription after excluding genes that are not specific for transcriptional regulation. **(D)** Western blot analysis of NUF2, KDM2A, and PCGF6 protein expression in 769-P and ACHN cells transfected with the si-NUF2 or si-NC for 48 h, with GAPDH as the internal control. **(E)** Schematic structure of the design for the ChIP assay of the *HMGA2* promoter. TSS, transcription start site. **(F, G)** 769-P cells were transfected with si-NUF2 or si-NC for 48 h. ChIP analysis of KDM2A, RNA Pol II, H3K36me, H3K36me2, and H3K36me3 enrichment in the *HMGA2* gene promoter; ChIP enrichment was measured using real-time PCR, normalized to the input DNA. Error bars represent the SEM from three independent experiments. **p < 0.01 *vs*. si-NC, *p < 0.05 *vs*. si-NC. **(H)** Western blot analysis of KDM2A and HMGA2 protein expression in 769-P and ACHN cells transfected with the si-KDM2A or si-NC for 48 h, with GAPDH as the internal control.

Next, we examined the molecular mechanism underlying NUF2 regulating HMGA2. 176 overlapping DEGs between siRNA-NUF2 and the negative control groups in 769-P and ACHN cells were screened out (Figure 5A), and were used to perform Gene Ontology (GO) analysis. The results showed that DEGs were mainly enriched in the process of transcriptional regulation (Figure 5B). The chord diagram further showed the GO terms related to transcriptional processes, including transcription, regulation of transcription, transcription factor activity, and DNA binding, with their 26 genes specifically involved in transcription (Figure 5C). Interestingly, we found seven genes associated with chromosome remodeling and gene transcription: PCGF6, KDM2A, SETD7, BRPF1, GATAD2A, BAHD1 and PRMT6 (Figure 5C). Among these, PCGF6 and KDM2A were both enriched in 3 GO terms. Specifically, NUF2 knockdown for 48 h sharply increased KDM2A protein level, but did not affect PCGF6 expression in 769-P and ACHN cells (Figure 5D). Consistently, further ChIP assay showed that NUF2 depletion enhanced KDM2A occupancy on* HMGA2* promoter, but decreased RNA Pol II enrichment in cells (Figure 5F). Since KDM2A has been reported to catalyze H3K36 demethylation to play an epigenetic regulatory role in target genes 21-23, we also examined H3K36 methylation levels and found that the enrichment of H3K36me2, which was usually associated with gene activation, at the *HMGA2* promoter was significantly decreased in NUF2-depleted cells, but the levels of H3K36me and H3K36me3 failed to detect a significant difference (Figure 5G). Moreover, knockdown of KDM2A by siRNA indeed increased expression of HMGA2 in 769-P and ACHN cells (Figure 5H). Therefore, we concluded that NUF2 regulates KDM2A-mediated H3K36me2 demethylation in *HMGA2* promoter region, consequently activates *HMGA2* transcription.

### NUF2-HMGA2 axis could be regarded as a prognostic biomarker in ccRCC

**Figure 6:**
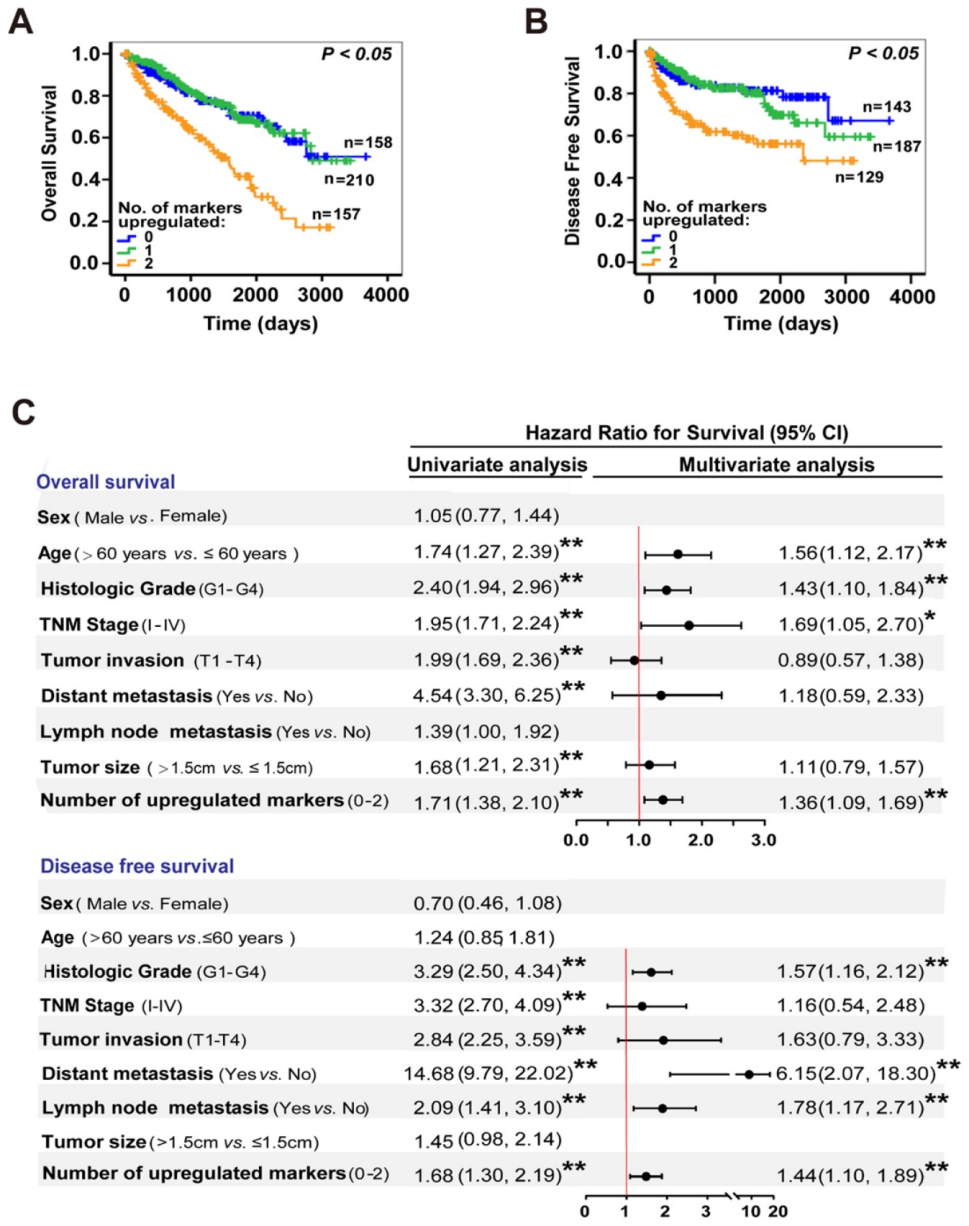
** NUF2-HMGA2 axis is an attractive candidate as a prognostic biomarker of ccRCC. (A, B)** Kaplan-Meier analysis of overall survival (A) and disease-free survival (B) for ccRCC patients based on the number of upregulated molecular markers; NUF2 and HMGA2 expression was stratified by the individual medians by RNA-sequencing data from TCGA dataset, and the patients were divided into three groups as indicated. **(C)** Univariate and multivariate Cox regression analysis of clinicopathologic variables and the number of upregulated markers associated with survival in patients with ccRCC in TCGA database. **p < 0.01, *p < 0.05.

Finally, we explored the clinical importance of the NUF2-HMGA2 axis in ccRCC patients. We generated a panel combining NUF2 and HMGA2 to predict ccRCC prognosis. Kaplan-Meier survival analysis and the log-rank test indicated that patients with both markers increased showed the shortest OS and DFS, while there was no difference between the group of only one marker was up-regulated and the group with NUF2 & HMGA2 both low expression (Figure 6A, 6B). Further analysis in Figure S4 shown that on the basis of high expression of NUF2, when HMGA2 was highly expressed at the same time, the survival condition of patients was the worst; However, only when NUF2 was highly expressed but HMGA2 was low expressed, there was no significant difference in the survival time between this group and the patients with low expression of NUF2; At the same time, when NUF2 was low expressed, whether HMGA2 was high expressed or not, there was no significant difference in the impact on the patient's survival compared with the NUF2 low expression group, which indicated that the effect of NUF2 high expression group on the prognosis of patients may come from the joint effect of NUF2 and HMGA2. Moreover, Cox regression multivariate analysis further illustrated that the number of upregulated markers could serve as an independent predictor to evaluate the survival of ccRCC patients (OS: HR = 1.36, 95% CI: 1.09-1.69, *p* < 0.01; DFS: HR = 1.44, 95% CI: 1.10-1.89, *p* < 0.01; Figure 6C). Hence, the NUF2-HMGA2 axis may have potential as a prognostic biomarker for ccRCC.

## Discussion

Accumulating evidence has indicated that NUF2 expression is upregulated in a series of human cancers, including colorectal 14, stomach 14, oral cancers 24, breast cancer 25, and osteosarcoma 26, and that the depletion of NUF2 suppresses cancer cell growth. Additionally, NUF2 can be used as a molecular marker to predict tumor progression in oral cancer 24, hepatocellular carcinoma 27, and lung cancer 28, among other cancers 29. More importantly, the results from a phase I clinical trial of NUF2 peptide vaccination for castration-resistant prostate cancer showed that NUF2 peptide vaccine therapy could significantly maintain patient quality of life and extend survival 30. Together, these results highlight a specific role of NUF2 in tumor growth and progression, making it a potential and effective candidate for molecule-targeted therapy in many cancers. Although our previous study 16 reported that NUF2 is involved in long noncoding RNA CDKN2BAS1-mediated ccRCC progression, the exact biological role and clinical significance of NUF2 in ccRCC have not yet been fully elucidated. In the present study, our findings from loss- (siRNA oligos) and gain-of-function (overexpression) experiments revealed that NUF2 is elevated and promotes cell proliferation, migration, and invasion in ccRCC, as in other tumors. Increased NUF2 expression is associated with worse clinicopathological variables and shorter survival rates. Cox regression analysis confirmed that NUF2 could also serve as an independent predictor of poor progression in ccRCC.

Until now, most evidence has shown that NUF2 drives tumorigenesis mainly by regulating cell cycle progression and DNA replication 29, 31, which is consistent with the reported role of NUF2 in mediating kinetochore-microtubule attachment during cell division 11, 12. However, little is known about the direct target factors that mediate the biological effects of NUF2 in tumors, especially in ccRCC. In this study, we performed gene expression profiling analysis in NUF2 depleted ccRCC cells to identify the potential target genes regulated by NUF2. Among the 176 overlapping DEGs after NUF2 depleting in 769-P and ACHN cells, *HMGA2*, which serves as an oncogene to promote the progression of various tumors 32, was the most downregulated gene in both cells. Therefore, we first and only explored the role of *HMGA2* in NUF2-driven ccRCC, and could not rule out that NUF2 could affect ccRCC progression through other downstream genes. Our following experiments prove that NUF2 plays a role in ccRCC cell proliferation, migration, and invasion, at least partly through HMGA2. Mechanistically, NUF2 could suppress KDM2A expression and affect its occupancy on *HMGA2* promoter region, which involves enhanced chromatin accessibility at promoter associated with increased H3K36me2 levels, thereby stimulating *HMGA2* transcription in ccRCC cells. In particular, NUF2 knockdown did not affect the enrichment of H3K36me and H3K36me3 in the* HMGA2* promoter region in our study. This could be supported by the report that KDM2A, a Jumonji C domain‑containing demethylase, specifically demethylates the H3K36me2 and exerts little or no activity on H3K36me and H3K36me3 33. Thus, our study contributes to further understanding the specific molecular mechanisms underlying NUF2-mediated ccRCC progression.

HMGA2, a member of the high-mobility group family, is regarded as a structural transcription factor that binds to the minor groove of DNA and causes it to bend, thereby contributing to transcriptional regulation of target genes 34. HMGA2 is highly expressed in embryonic stem cells during embryogenesis, whereas its expression is decreased during postembryonic development 32. Nevertheless, HMGA2 is abnormally re-expressed in nearly all human cancers, where it acts as an oncofetal protein that has been reported to induce cancer cell proliferation by promoting cell cycle and inhibiting apoptosis, as well as facilitating epithelial-mesenchymal transition by activating the PI3K/AKT/mTOR/NFKB, TGFβ/SMAD, MAPK/ERK, and STAT3 pathways 32. These findings explain why NUF2 could accelerate both ccRCC cell proliferation and invasion via HMGA2 in this study. In addition, the results of our study and that of others 35 showed that HMGA2 levels in ccRCC tissues could be used as a predictor of clinical prognosis. Therefore, these data, together with previous reports 35-38, further support the role of HMGA2 in ccRCC, and provide more experimental evidence and a theoretical basis for using HMGA2 as a therapeutic target in patients with ccRCC.

Finally, we comprehensively discussed the value of the NUF2-HMGA2 axis as a prognostic biomarker in ccRCC. Our data revealed that the joint action of NUF2 and HMGA2 leads to a shorter patient's survival, and the number of upregulated markers was positively correlated with poor survival and acted as an independent predictor of survival probability, indicating that the NUF2-HMGA2 axis has a cumulative prognostic predictive capability. Collectively, these results highlight the prognostic biomarker potential of the NUF2-HMGA2 axis in ccRCC, although it needs to be confirmed in a larger independent sample cohort.

In summary, the findings of the present study demonstrate that NUF2 is overexpressed and correlates with poor prognosis in ccRCC. It also acts as a potential oncogene to promote the proliferation, migration, and invasion of ccRCC cells through epigenetic activation of *HMGA2* transcription by affecting the recruitment of KDM2A to regulate H3K36me2 modification in the promoter region. Overall, the NUF2-HMGA2 axis was identified as a novel interaction regulating tumorigenesis and progression in ccRCC, and thus can be an ideal therapeutic target and a promising prognostic indicator for ccRCC.

## Figures and Tables

**Figure 7:**
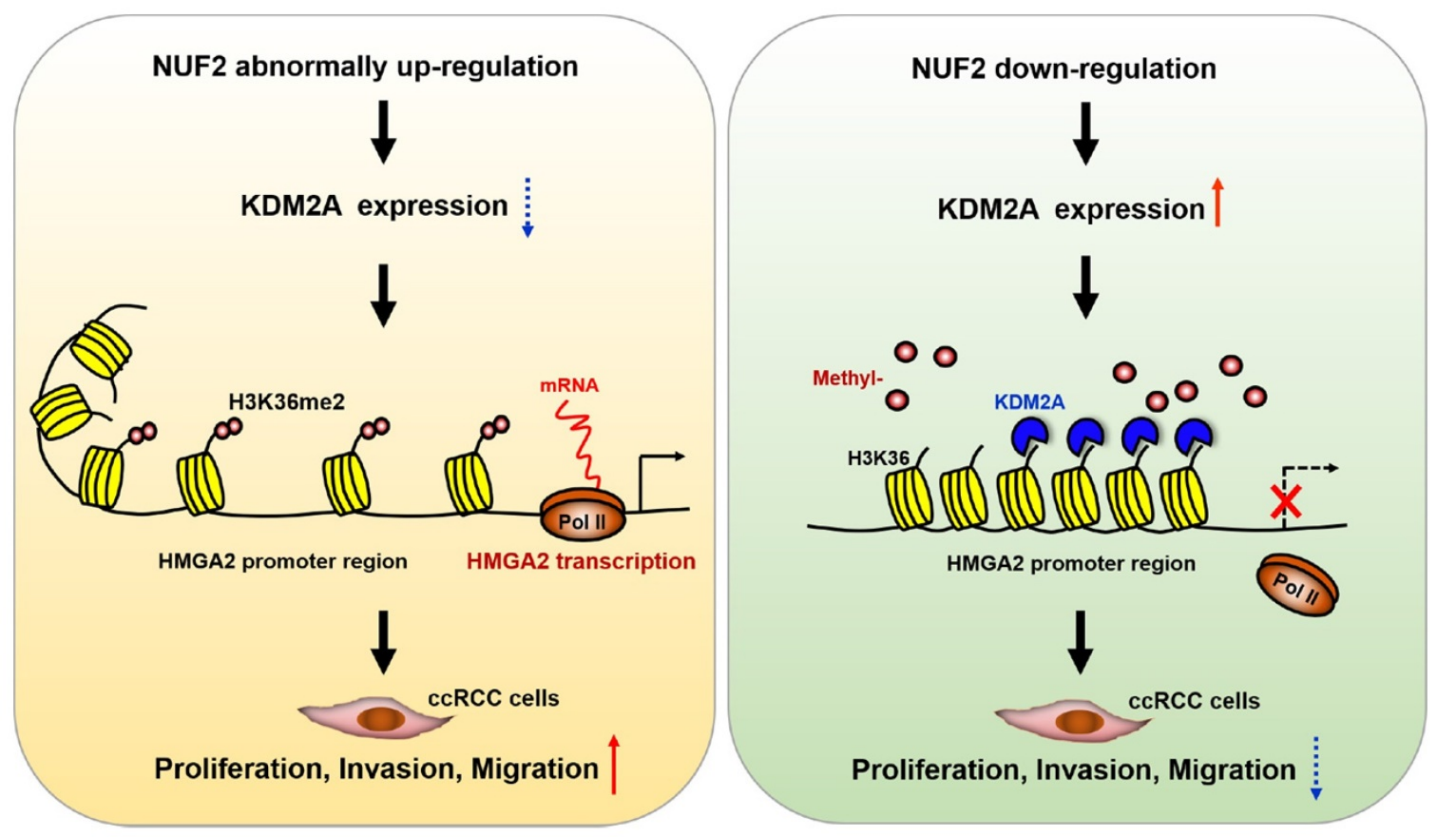
** Schematic diagram demonstrating the molecular mechanism underlying NUF2 in ccRCC.** NUF2 is abnormally overexpressed in ccRCC; it could suppress KDM2A expression and affect its occupancy on *HMGA2* promoter region, which involves enhanced chromatin accessibility at promoter associated with increased H3K36me2 levels, thereby stimulating *HMGA2* transcription and further promoting cell proliferation, migration, and invasion.

**Table 1:** Univariate and multivariate Cox regression analyses of clinical factors associated with survival in ccRCC.

Variables	Univariate analysis				Multivariate analysis		
	HR	95%CI	*P* value		HR	95%CI	*P* value
**Overall Survival (Tissue microarray)**							
Sex (Male *vs.* Female)	1.084	(0.526, 2.233)	0.828				
Age (>60 years *vs. ≤*60 years )	3.014	(1.385, 6.558)	*0.005*		5.927	(2.374, 14.797)	*<0.001*
Histologic Grade (G3-4 *vs.* G1-2)	5.321	(2.564, 11.045)	*<0.001*		4.488	(2.014, 9.998)	*<0.001*
TNM Stage (III-IV* vs.* I-II)	5.306	(2.129, 13.223)	*<0.001*		4.367	(1.497, 12.746)	*0.007*
Tumor size (>4cm *vs. ≤*4cm)	1.687	(0.808, 3.522)	0.164				
NUF2 protein level	1.016	(1.001, 1.032)	*0.042*		1.02	(1.002, 1.039)	*0.031*
**Overall Survival (TCGA database)**							
Sex (Male *vs.* Female)	1.052	(0.768, 1.442)	0.752				
Age (>60 years *vs. ≤*60 years )	1.742	(1.271, 2.386)	*0.001*		1.679	(1.205, 2.338)	*0.002*
Histologic Grade (G1-G4)	2.398	(1.940, 2.964)	*<0.001*		1.375	(1.064, 1.776)	*0.015*
TNM Stage (I-IV)	1.953	(1.707, 2.236)	*<0.001*		1.549	(0.971, 2.472)	0.066
Tumor invasion (T1-T4)	1.992	(1.685, 2.355)	*<0.001*		0.950	(0.616, 1.465)	0.816
Distant metastasis (Yes *vs.*No)	4.544	(3.303, 6.251)	*<0.001*		1.238	(0.628, 2.441)	0.537
Lymph node metastasis (Yes *vs.*No)	1.385	(1.000, 1.918)	0.050				
Tumor size (>1.5cm *vs. ≤*1.5cm)	1.675	(1.213, 2.312)	*0.002*		1.067	(0.758, 1.503)	0.709
NUF2 mRNA level	1.590	(1.396, 1.811)	*<0.001*		1.280	(1.101, 1.488)	*0.001*
**Disease Free Survival (TCGA database)**							
Sex (Male *vs.* Female)	0.704	(0.461, 1.075)	0.104				
Age (>60 years *vs. ≤*60 years)	1.238	(0.845, 1.814)	0.273				
Histologic Grade (G1-G4)	3.294	(2.498, 4.344)	*<0.001*		1.521	(1.115, 2.074)	*0.008*
TNM Stage (I-IV)	3.320	(2.696, 4.088)	*<0.001*		0.914	(0.420, 1.993)	0.822
Tumor invasion (T1-T4)	2.843	(2.252, 3.589)	*<0.001*		2.034	(0.979, 4.225)	0.057
Distant metastasis (Yes *vs.* No)	14.678	(9.785, 22.019)	*<0.001*		7.511	(2.485, 22.702)	*<0.001*
Lymph node metastasis (Yes *vs.* No)	2.093	(1.414, 3.100)	*<0.001*		1.889	(1.243, 2.871)	*0.003*
Tumor size (>1.5cm *vs. ≤*1.5cm)	1.45	(0.981, 2.144)	0.062				
NUF2 mRNA level	1.635	(1.379, 1.938)	*<0.001*		1.227	(1.016, 1.482)	*0.034*

**Abbreviations:** HR: hazard ratio, CI: confidence interval, TNM: Tumor node metastasis.

## Abbreviations

ccRCC: clear cell renal cell carcinoma
NUF2: Ndc80 kinetochore complex component
HMGA2: high-mobility group AT-hook 2
KDM2A: lysine demethylase 2A
H3K36me2: histone H3 lysine 36 di-methylation
TCGA: The Cancer Genome Atlas
siRNAs: small interfering RNAs
PCGF6: polycomb group factor 6
DEGs: differentially expressed genes
ChIP: chromatin immunoprecipitation
RNA Pol II: RNA Polymerase II
H3K36me: histone H3 lysine 36 mono methylation
H3K36me3: histone H3 lysine 36 trimethylation
OS: overall survival
DFS: disease-free survival
GO: Gene Ontology
HR: hazard ratio
CI: confidence interval
TNM stage: tumor node metastasis stage

## Supplementary Material

Supplementary figures and tables.

Supplementary data - RNA-seq DEGs and GO analysis.

Supplementary data - The original clinical data from TCGA.
