# Supplementary material for: NUF2 Drives Clear Cell Renal Cell Carcinoma by Activating HMGA2 Transcription through KDM2A-mediated H3K36me2 Demethylation
Source: Int J Biol Sci. 2022 May 16;18(9):3621–35. doi: 10.7150/ijbs.70972 (PMC9254462; doi:10.7150/ijbs.70972)
Supplement: Supplementary file 1 — Supplementary figures and tables. [file ijbsv18p3621s1.pdf]

Supplementary Materials

Supplementary Figures and Tables

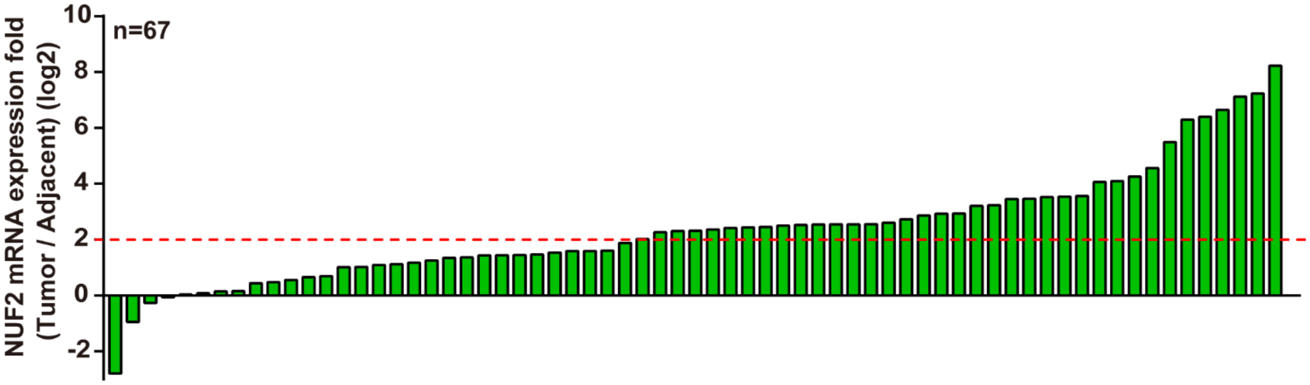

Figure S1. Expression of NUF2 in ccRCC tissues. qRT-PCR analysis of NUF2 expression in ccRCC tissues (Tumor) and corresponding tumor-adjacent normal tissues (Adjacent), with β-actin was used as the internal control. The ratio of NUF2 expression level in Tumor to Adjacent was measured to analyze the fold change for each patient, n=67.

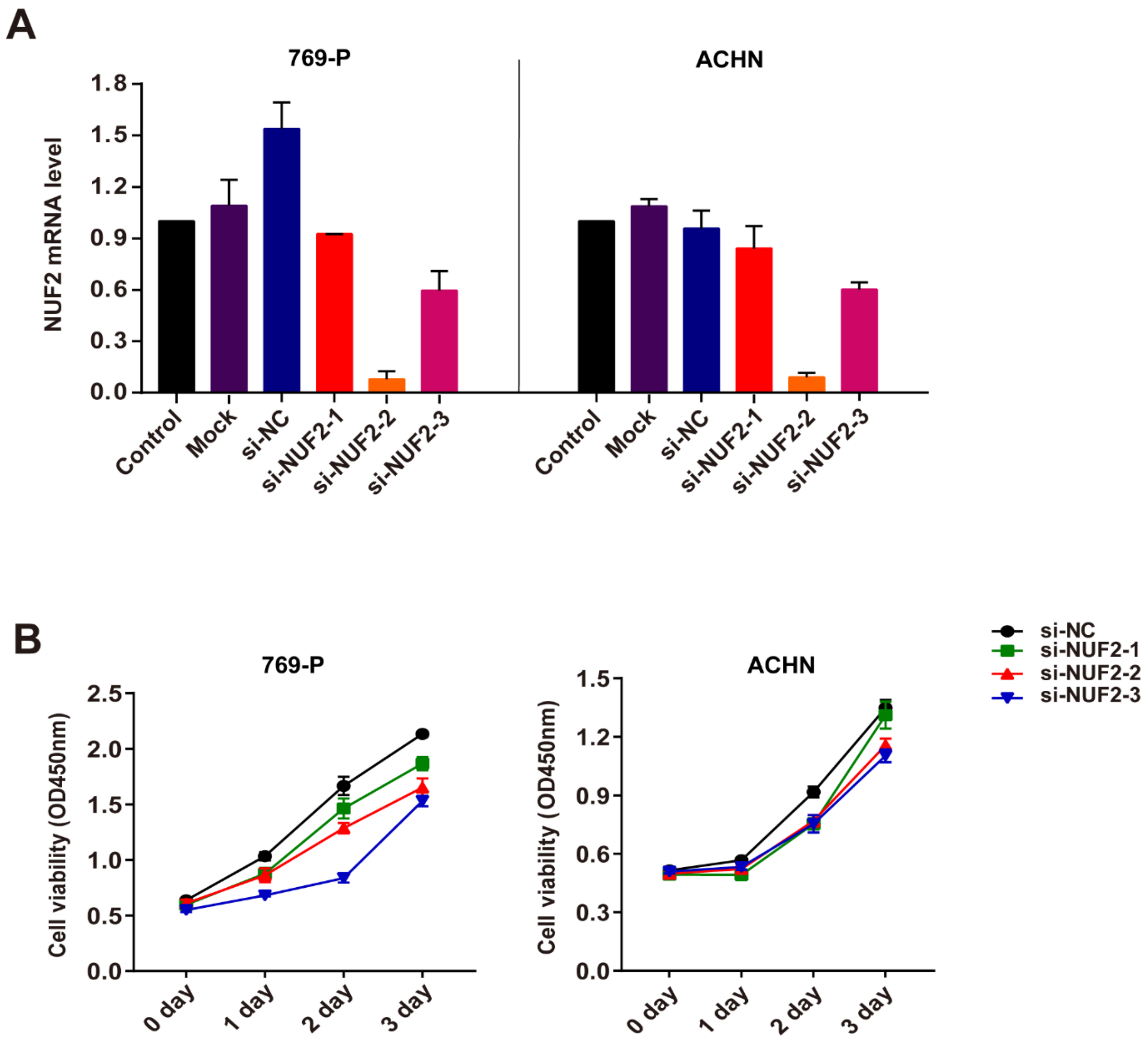

Figure S2. Depletion of NUF2 suppresses ccRCC cell viability. 769-P and ACHN cells transfected with siRNAs (siRNA1, siRNA2, siRNA3) against NUF2 for 48 h, respectively. **(A)** qRT-PCR analysis of NUF2 in 769-P and ACHN cells, with β-actin was used as the internal control. **(B)** Cell Counting Kit-8 assay was used to assess 769-P and ACHN cell viability. Based on these pre-experimental results, we will mix siRNA2 and siRNA3 together (si-NUF2) for NUF2 knockdown in subsequent experiments. Error bars represent the SEM.

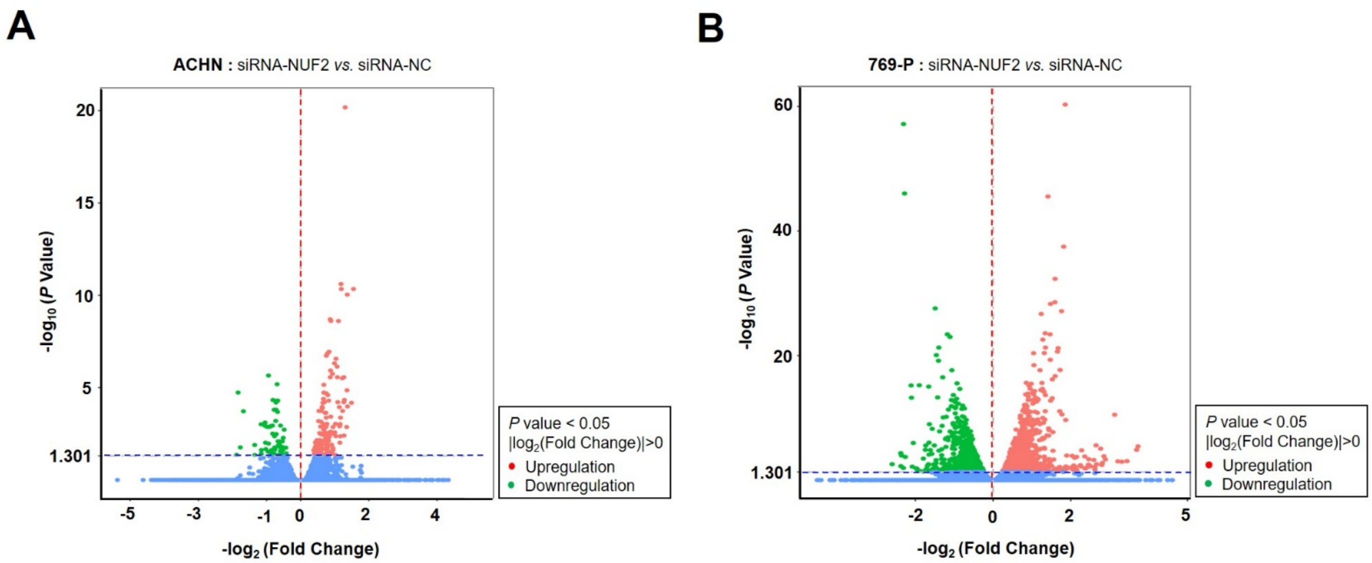

Figure S3. The regulated genes of NUF2 in ccRCC cells. Volcano diagram depicting the differentially expressed genes from the RNA sequencing in ACHN **(A)** and 769-P **(B)** cells transfected with siRNA-NUF2 or siRNA-NC, respectively. Red represents upregulated genes, blue represents downregulated genes, blue represents no-signification.

A

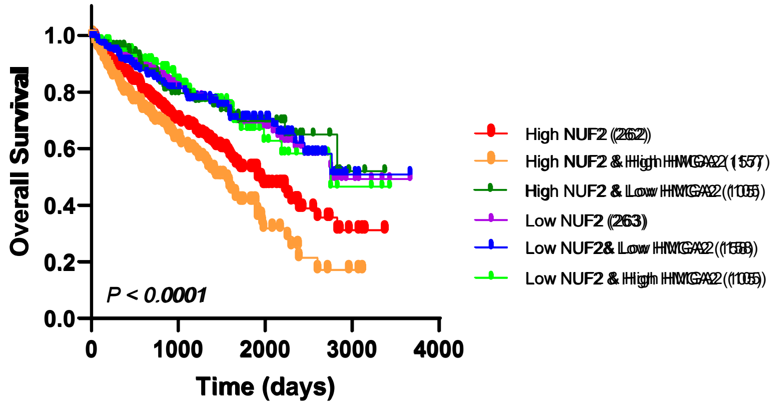

B

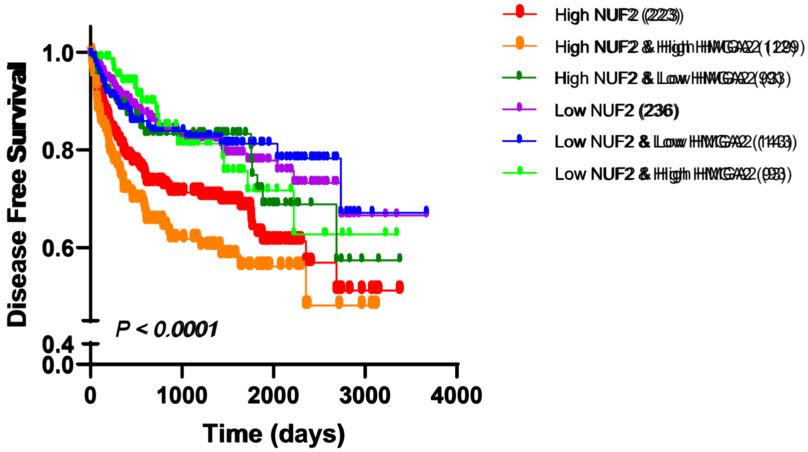

Figure S4. NUF2 and HMGA2 synergistically promote malignant phenotypes with higher mortality. Kaplan–Meier analysis showed the overall survival (OS) **(A)** and disease-free survival (DFS) **(B)** in ccRCC patients based on the expression of NUF2 and HMGA2; the high and low expression groups of NUF2 and HMGA2 were respectively divided by their median mRNA expression levels in TCGA dataset. Firstly, the patients were divided into two groups according to the high and low expression level of NUF2 gene, and then on this basis, they were further divided into groups according to HMGA2 gene expression. Patients were divided into six groups as indicated.

Table S1. Correlation between the expression of NUF2 / HMGA2 and clinicopathological features in ccRCC (TCGA database).

Variable	Categorization	No. of analysis (%)	NUF2 level §	*P* value	HMGA2 level §	*P* value
**Age**				0.959		0.537
	≤60 years	259 (49.1%)	5.06 ± 1.19		1.89 ± 1.82	
	＞60 years	269 (50.9%)	5.06 ± 1.24		2.00 ± 2.35	
**Sex**				*0.020*		0.378
	Male	341 (64.6%)	5.15 ± 1.24		2.01 ± 2.07	
	Female	187 (35.4%)	4.89 ± 1.14		1.84 ± 2.18	
**Tumor size**				*0.027*		0.179
	≤1.5cm	255 (54.0%)	4.98 ± 1.22		1.83 ± 1.95	
	＞1.5cm	217 (46.0%)	5.22 ± 1.16		2.10 ± 2.35	
**Histologic Grade**				*<0.001*		*<0.001*
	G1	13 (2.5%)	4.37 ± 0.71		1.11 ± 0.96	
	G2	228 (43.8%)	4.78 ± 1.01		1.59 ± 1.54	
	G3	204 (39.2%)	5.14 ± 1.17		1.85 ± 185	
	G4	75 (14.5%)	5.83 ± 1.52		3.36 ± 3.38	
**TNM Stage**				*<0.001*		*<0.001*
	I	264 (50.0%)	4.78 ± 1.03		1.61 ± 1.48	
	II	57 (10.8%)	4.84 ± 1.28		1.60 ± 1.77	
	III	126 (23.9%)	5.22 ± 1.15		2.21 ± 2.41	
	IV	81 (15.3%)	5.86 ± 1.41		2.88 ± 3.06	
**Tumor invasion**				*<0.001*		*<0.001*
	T1	269 (50.9%)	4.78 ± 1.04		1.62 ± 1.48	
	T2	69 (13.1%)	4.94 ± 1.24		1.52 ± 1.66	
	T3	179 (33.9%)	5.39 ± 1.19		2.35 ± 2.56	
	T4	11 (2.1%)	7.19 ± 1.93		6.10 ± 3.77	
**Distant metastasis**				*<0.001*		*0.007*
	No	422 (84.2%)	4.94 ± 1.16		1.83 ± 1.96	
	Yes	79 (15.8%)	5.79 ± 1.30		2.74 ± 2.80	
**Lymph node metastasis**				*<0.001*		*<0.001*
	No	388 (74.5%)	4.94 ± 1.13		1.65 ± 1.67	
	Yes	133 (25.5%)	5.46 ± 1.32		2.84 ± 2.92	

§Data were shown as mean ± SD.

Table S2. Correlation between the protein level of NUF2 and clinicopathological features in ccRCC (Tissue microarray).

		NUF2 protein (Immunoreative score) §	*P* value
**Age**			0.325
≤60 years	50 (55.6%)	3.92 ± 1.31	
＞60 years	38 (42.2%)	3.66 ± 1.12	
**Sex**			0.831
Male	59 (65.6%)	3.78 ± 1.26	
Female	31 (34.4%)	3.84 ± 1.21	
**Tumor size**			0.45
≤4 cm	37 (41.1%)	3.92 ± 1.16	
＞4 cm	53 (58.8%)	3.72 ± 1.29	
**Histologic Grade**			*0.001*
G1-2	63 (70.0%)	3.52 ± 1.15	
G3-4	27 (30.0%)	4.44 ± 1.22	
**TNM Stage**			*0.024*
I-II	79 (87.8%)	3.72 ± 1.22	
III-IV	8 (8.9%)	4.75 ± 1.04	

§Data were shown as mean ± SD; TNM: Tumor node metastasis.

Table S3. Univariate Cox regression analyses of clinical factors associated with survival in ccRCC (TCGA database).

Variables	Overall Survival			Disease Free Survival		
	**HR**	**95%CI**	***P* value**	**HR**	**95%CI**	***P* value**
**Sex** (Male *vs.* Female)	1.052	(0.768, 1.442)	0.752	0.704	(0.461, 1.075)	0.104
**Age** (＞60 years *vs.*≤60 years)	1.742	(1.271, 2.386)	*0.001*	1.238	(0.845, 1.814)	0.273
**Histologic Grade** (G1-G4)	2.398	(1.940, 2.964)	*<0.001*	3.294	(2.498, 4.344)	*<0.001*
**TNM Stage** (I-IV)	1.953	(1.707, 2.236)	*<0.001*	3.320	(2.696, 4.088)	*<0.001*
**Tumor invasion** (T1-T4)	1.992	(1.685, 2.355)	*<0.001*	2.843	(2.252, 3.589)	*<0.001*
**Distant metastasis** (Yes *vs.* No)	4.544	(3.303, 6.251)	*<0.001*	14.678	(9.785, 22.019)	*<0.001*
**Lymph node metastasis** (Yes *vs.* No)	1.385	(1.000, 1.918)	0.050	2.093	(1.414, 3.100)	*<0.001*
**Tumor size** (＞1.5cm *vs.*≤1.5cm)	1.675	(1.213, 2.312)	*0.002*	1.45	(0.981, 2.144)	0.062
**HMGA2 mRNA level**	1.202	(1.140, 1.268)	*<0.001*	1.198	(1.115, 1.287)	*<0.001*

Abbreviations: HR: hazard ratio; CI: confidence interval.
